# Thoracic Spinal Epidural Abscess After Spinal Cord Stimulation Trial With Cord Compression but Preserved Neurologic Function: A Rehabilitation Case

**DOI:** 10.7759/cureus.109630

**Published:** 2026-05-25

**Authors:** James Rodriguez, Steven Hoffman

**Affiliations:** 1 Physical Medicine and Rehabilitation, Larkin Community Hospital, South Miami, USA; 2 Pain Management, Larkin Community Hospital, South Miami, USA

**Keywords:** rehabilitation, scs, scs trial, spinal cord stimulation (scs), spinal cord stimulator, spinal epidural abscess, thoracic epidural abscess

## Abstract

Spinal cord stimulation (SCS) is an effective therapeutic option for refractory neuropathic pain, but infectious complications, particularly epidural abscess, are rare and carry high morbidity. Evidence describing the inpatient rehabilitation course following urgent surgical decompression in patients with radiographic cord compression but largely preserved neurologic function remains limited, and standardized functional outcomes are seldom reported. We present the case of an 84-year-old woman with severe dextroscoliosis, kyphosis, and chronic axial back pain who underwent an outpatient SCS trial. Five days later, on the day of lead removal, she presented with fever (maximum temperature 38.5 °C), tachycardia, leukocytosis (white blood cell count 11.58 K/μL), markedly elevated inflammatory markers (C-reactive protein 312 mg/L on repeat testing after an initial erroneously high reading, procalcitonin 10.11 ng/mL, and erythrocyte sedimentation rate 63 mm/hr), and urinary retention. Thoracic MRI demonstrated a dorsal-predominant epidural collection from T8-T12 with cord compression and adjacent paraspinal cellulitis. She underwent urgent T8-T12 decompressive laminectomies and was treated for methicillin-resistant *Staphylococcus aureus* bacteremia with daptomycin. Following acute care, she completed a 23-day inpatient rehabilitation admission. Functional progress was tracked with the seven-point functional independence measure (FIM). The FIM motor sub-score improved from 47 on admission to 68 on discharge (a 21-point gain). Pain, monitored with the Numeric Rating Scale (NRS), decreased from 6/10 on admission to 2/10 on discharge with a multimodal analgesic regimen. By discharge, she ambulated 300 feet independently without an assistive device and was modified independent on 12 steps. This case suggests that inpatient rehabilitation may be safely delivered to selected older adults with radiographically severe but clinically preserved post-SCS epidural abscess. It also supports the routine reporting of FIM-based outcomes in this rare scenario.

## Introduction

Spinal cord stimulation (SCS) is a well-established neuromodulatory therapy for refractory neuropathic pain, including failed back surgery syndrome, complex regional pain syndrome, and painful diabetic neuropathy. Reported complications include lead migration, hardware failure, and infection; surgical site infection occurs in approximately 2.5-4.5% of cases, while deep infections such as epidural abscess remain rare but clinically high-risk [1].

Epidural abscess in the context of an SCS trial is exceedingly uncommon and has been reported in only a few cases [2-4]. Risk factors include advanced age, immunosuppression, diabetes mellitus, obesity, prolonged trial duration, and breaks in sterile technique. The classic triad of fever, back pain, and neurologic deficit is often incomplete at presentation, and progression to cord compression can be rapid.

Although surgical and antimicrobial management of spinal epidural abscess is well described, the rehabilitation literature offers limited guidance for patients who retain a largely intact neurologic examination despite radiographic cord compression. Standardized rehabilitation outcome measures, such as the functional independence measure (FIM), are seldom reported in this population, limiting comparability across studies. This report describes the inpatient rehabilitation course of an 84-year-old woman who developed a thoracic epidural abscess and cord compression following an SCS trial, with functional recovery quantified using the FIM and pain quantified using the Numeric Rating Scale (NRS), to add to the limited literature guiding rehabilitation in this rare clinical scenario [5].

## Case presentation

Premorbid functional status

Prior to the index admission, the patient lived independently at home with her family. She was a community ambulator without an assistive device, fully independent in all basic activities of daily living (ADLs) and most instrumental ADLs (medication management, light cooking, and light housekeeping), and able to negotiate a 12-step entry staircase with a single rail. Chronic axial back pain (baseline NRS 4/10) had progressively limited her tolerance for community ambulation in the months preceding the SCS trial.

Initial presentation

This right-hand-dominant patient with a past medical history of bilateral breast cancer (stage Ia, T1a) status post bilateral lumpectomy (right 1991, left 2014) in remission; chronic right upper extremity lymphedema; hypertension; chronic cytopenia; glaucoma; severe dextroscoliosis with kyphosis; and chronic back pain presented for an outpatient SCS trial. Trial leads were placed on September 10, 2025, and removed on September 15, 2025, after a five-day trial.

On the same day as lead removal, the patient presented to the emergency department with fever, generalized weakness, and urinary retention. Vital signs were notable for a maximum temperature (Tmax) of 38.5 °C and tachycardia. Initial laboratory workup is summarized in Table 1. The initial automated CRP result was “>1250 mg/L,” prompting clinical concern for a reporting or transcription artifact given the assay’s upper reporting limit. A repeat quantitative CRP on a redrawn sample returned a value of 312 mg/L, which was used for subsequent clinical decision-making. Blood cultures grew methicillin-resistant *Staphylococcus aureus* (MRSA).

**Table 1:** Initial laboratory and vital sign findings on presentation with reference ranges * The initial CRP assay returned a flagged result above the upper reporting limit of the analyzer. The value was re-verified on a freshly drawn specimen, yielding 312 mg/L. The flagged result is preserved in the medical record, but the verified repeat value was used for clinical decision-making. MRSA: methicillin-resistant *Staphylococcus aureus*, WBC: white blood cell, ESR: erythrocyte sedimentation rate, CRP: C-reactive protein

Parameter	Patient value	Reference range	Interpretation
Maximum temperature (Tmax)	38.5 °C	36.5-37.5 °C	Elevated (febrile)
Heart rate	Tachycardic	60-100 beats/min	Elevated
WBC count	11.58 K/μL	4.5-11.0 K/μL	Leukocytosis
Potassium (K⁺)	3.3 mmol/L	3.5-5.0 mmol/L	Hypokalemia
Sodium (Na⁺)	132 mmol/L	135-145 mmol/L	Mild hyponatremia
Serum creatinine	1.37 mg/dL	0.6-1.1 mg/dL (female)	Elevated
ESR	63 mm/hr	0-20 mm/hr (female)	Markedly elevated
Procalcitonin	10.11 ng/mL	<0.10 ng/mL	Markedly elevated; consistent with bacterial sepsis
CRP*	312 mg/L on repeat (initial assay flagged >1250 mg/L)	<10 mg/L	Markedly elevated; initial value re-verified to exclude transcription/assay artifact
Blood cultures	Positive for MRSA	No growth	MRSA

Thoracic MRI with contrast on September 16, 2025, demonstrated a new dorsal-predominant epidural collection extending from T8-T12 with associated spinal cord compression and adjacent paraspinal soft-tissue cellulitis. Concurrent CT of the abdomen and pelvis revealed constipation, possible mild colitis, a small splenic infarct, a distended gallbladder, a small left pleural effusion, and small nodular pulmonary infiltrates concerning for pneumonia versus septic emboli. A para-isopropyl iminodiacetic acid scan demonstrated a gallbladder ejection fraction of 8%, consistent with biliary dyskinesia. Lumbar imaging confirmed severe dextroscoliosis and moderate L4-L5 canal stenosis. Transthoracic echocardiogram (TTE) showed no evidence of endocarditis.

Surgical and acute medical management

On September 17, 2025, the patient underwent urgent T8-T12 decompressive laminectomies with evacuation of purulent material from the paraspinal musculature and epidural space. Intraoperative findings confirmed frank purulence, and the surgical bed was irrigated with an antibiotic solution. A 15-round Blake JP drain was placed, and the wound was closed in layers with Vicryl sutures and skin staples. Operative cultures also grew MRSA, consistent with blood culture results [1].

Antimicrobial therapy, dosed for renal function and serum levels, included intravenous daptomycin 6 mg/kg every 24 hours (administered via a right subclavian central venous catheter for a planned six-week course completed October 29, 2025), with adjunctive intravenous cefepime 2 g every 12 hours and intravenous vancomycin 15 mg/kg every 12 hours (titrated to trough) during the initial empiric phase and oral doxycycline 100 mg twice daily during transitions, as directed by the infectious disease team. Multimodal analgesia consisted of gabapentin 300 mg orally three times daily, scheduled acetaminophen 650 mg orally every six hours, morphine 2-4 mg intravenously every 4 hours as needed for severe breakthrough pain, and tramadol 50 mg orally every 6 hours as needed for moderate pain. Pharmacologic venous thromboembolism prophylaxis with enoxaparin 40 mg subcutaneously daily and a structured bowel regimen (docusate 100 mg twice daily and senna 8.6 mg nightly) was initiated and maintained throughout admission.

Admission to inpatient rehabilitation (October 7, 2025)

**Figure 1:**
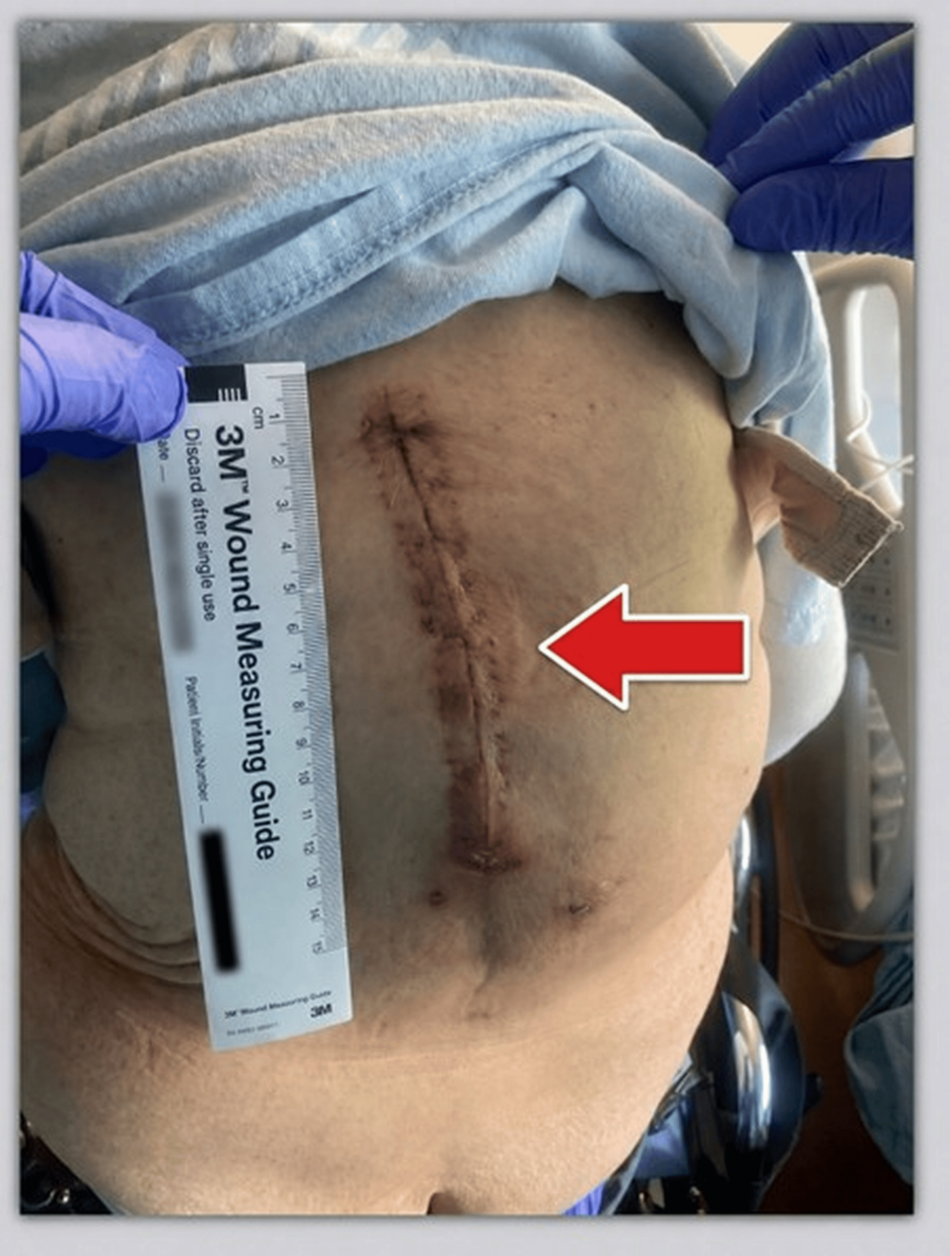
Midline thoracic surgical incision Healing midline thoracic surgical incision (red arrow) following T8–T12 decompressive laminectomies for evacuation of a MRSA epidural abscess, photographed during the inpatient rehabilitation admission. The incision is being measured with a sterile 3M Wound Measuring Guide and shows no erythema, dehiscence, drainage, or fluctuance, consistent with appropriate post-operative healing. Handwritten identifiers on the measuring guide have been redacted to protect patient privacy. Image obtained and used with the patient's written informed consent. MRSA: methicillin-resistant *Staphylococcus aureus*

On October 7, 2025, the patient was admitted to inpatient rehabilitation. On admission, she was alert, oriented, and hemodynamically stable. Neurologic examination was largely unremarkable, with no hyperreflexia or clonus. Manual muscle testing of the bilateral lower extremities revealed hip flexors 3/5 bilaterally, with all other muscle groups 4/5; deep tendon reflexes were 2/4 throughout. Sensation was grossly intact, and there were no signs of myelopathy. Musculoskeletal examination was notable for a healing midline thoracic surgical incision without erythema, drainage, or fluctuance (Figure 1). Admission pain was 6/10 on NRS. She was placed on strict thoracic spine precautions (no bending, lifting, or twisting) and continued on her targeted antimicrobial regimen. Baseline FIM scores at admission are presented in Table 2.

Rehabilitation course

The rehabilitation plan was function-focused and emphasized progressive mobilization under spinal precautions, infection surveillance with serial monitoring of inflammatory markers, multimodal analgesia, and patient and family education. Physical therapy targeted bed mobility, sit-to-stand transfers, ambulation with progressive reduction of assistive devices, and graded core and lower-extremity strengthening within precautions. Occupational therapy focused on ADLs, upper-extremity strengthening within right upper extremity lymphedema precautions, and adaptive equipment training for safe self-care. Each discipline delivered at least 90 minutes per day, with the patient consistently tolerating ≥3 hours of combined skilled therapy daily, consistent with inpatient rehabilitation hospital (IRH)-level care [4].

The patient progressed steadily through her rehabilitation course. By October 22, 2025, she required only close supervision for toileting and ambulated 160 feet with a rolling walker. On October 23, 2025, she ambulated 160 feet with contact-guard assistance without a device. By October 27, 2025, she was independent for both lower- and upper-body dressing, modified independent for bathing and toileting, and ambulated 350 feet with close supervision. Intravenous daptomycin was completed on October 29, 2025. On October 30, 2025, the day of discharge, the surgical incision was completely healed, with no evidence of dehiscence or infection (Figure 1). She was independent in toileting, bathing, upper- and lower-body dressing, toilet transfers, and bed mobility; ambulated 300 feet independently without any assistive device; and was modified independent for elevation on 12 steps. Discharge pain had decreased to 2/10 on the NRS. No neurologic decline occurred during her IRH stay [3,4].

Functional outcomes

Functional status was operationalized using the seven-point FIM, where 7 = complete independence; 6 = modified independence (device, extra time, or safety considerations); 5 = supervision or set-up; 4 = minimal contact assistance (patient performs ≥75%); 3 = moderate assistance (50-74%); 2 = maximal assistance (25-49%); 1 = total assistance (<25%). Admission FIM scores were generated by the admitting interdisciplinary therapy team on October 7, 2025; discharge FIM scores reflect the final occupational and physical therapy assessments on October 30, 2025. A side-by-side comparison is presented in Table 2. Across the nine domains tracked, the patient improved by ≥1 FIM point in every category, with a FIM motor sub-score gain of 21 points (47 → 68). Pain improved from 6/10 to 2/10 on NRS over the same interval.

**Table 2:** FIM scores at admission and discharge from inpatient rehabilitation FIM scoring key: 7 = complete independence, 6 = modified independence (device, extra time, or safety considerations), 5 = supervision or set-up, 4 = minimal contact assistance (patient ≥75%), 3 = moderate assistance (patient 50-74%), 2 = maximal assistance (patient 25-49%), 1 = total assistance (patient <25%). FIM motor sub-score in this table is the sum of the nine routinely tracked motor domains (eating, grooming, bathing, upper-body dressing, lower-body dressing, toileting, bed-to-chair transfer, toilet transfer, and ambulation). Stairs are reported separately and not included in the motor sub-score. NRS: Numeric Rating Scale, FIM: functional independence measure

Functional task (FIM domain)	Admission (October 7, 2025)	Discharge (October 30, 2025)
Eating	6 - modified independent	6 - modified independent
Oral hygiene/grooming	5 - supervision/set-up	6 - modified independent
Upper-body dressing	4 - minimal contact assistance	7 - complete independence
Lower-body dressing	3 - moderate assistance	7 - complete independence
Toileting	4 - minimal contact assistance	7 - complete independence
Toilet transfer	4 - minimal contact assistance	7 - complete independence
Bed-to-chair transfer	4 - minimal contact assistance	7 - complete independence
Showering/bathing	3 - moderate assistance	7 - complete independence
Bed mobility	5 - supervision	7 - complete independence
Ambulation (level surface)	4 - minimal assistance, 160 ft with rolling walker	7 - independent, 300 ft, no device
Stairs (12 steps)	Not attempted (deferred for safety/spine precautions)	6 - modified independent, 12 steps
Wheelchair mobility	5 - supervision	7 - independent, 300 ft
FIM motor sub-score (sum, tracked domains)	47	68 (Δ +21)
Pain (NRS, 0-10)	6/10	2/10

## Discussion

This case describes the inpatient rehabilitation course of a patient who developed a rare and potentially catastrophic complication of SCS, a thoracic epidural abscess with radiographic cord compression, yet retained a largely intact neurologic examination. The principal rehabilitation problem was not the management of established neurologic deficits but the safe restoration of function in a high-risk older adult recovering from a multilevel laminectomy, ongoing treatment of MRSA bacteremia, and strict thoracic spine precautions.

SCS-related infection is reported in approximately 2.5-4.5% of cases [1]. Epidural abscess is rarer still, and the literature describing its presentation during or shortly after a percutaneous trial is limited to case reports and small series [2,3]. As Mukhdomi and colleagues described, epidural extension of surgical site infection from an SCS trial may evolve subacutely and present after lead removal, when clinical suspicion can be inappropriately low [4]. In our patient, symptoms culminated on the day of lead removal, underscoring the need for continued vigilance after hardware withdrawal. Recognition of the constellation of fever, urinary retention, and elevated inflammatory markers, followed by rapid MRI and surgical decompression, plausibly contributed to her favorable neurologic trajectory. However, as a single case report, we cannot establish causation.

From a physical medicine and rehabilitation (PM&R) perspective, this case adds to the small body of literature on post-decompression rehabilitation in patients who are “radiographically positive but neurologically preserved.” Importantly, the magnitude of functional recovery was quantified using the FIM, which is reported only inconsistently in the existing SCS-abscess literature. Across the nine tracked motor domains, the patient improved by a total of 21 FIM points (Table 2), with the largest gains seen in lower-body dressing, bathing, and ambulation. Pain, tracked daily with the NRS, decreased from 6/10 on admission to 2/10 at discharge, consistent with effective multimodal analgesia and progressive de-escalation of opioid use. These standardized improvements between pre- and post-rehabilitation assessment provide objective evidence of recovery and address a limitation highlighted in similar case reports.

Several elements of her course warrant emphasis. First, a multidisciplinary infection-monitoring strategy, daily examination of the surgical incision (Figure 1), trending of inflammatory markers, and protocolized assessment for new neurologic findings, was associated with safe intensification of therapy. Second, the multimodal analgesic regimen (gabapentin 300 mg three times daily, scheduled acetaminophen 650 mg every six hours, and opioid-sparing as-needed morphine and tramadol) appeared to enable participation in ≥3 hours of skilled therapy per day, consistent with IRH-level care [4]. Third, although her hip flexors were initially 3/5 bilaterally, likely reflecting a combination of deconditioning, pain, and post-operative changes rather than a true upper motor neuron lesion, targeted strengthening within precautions was followed by functional ambulation without an assistive device by discharge.

This case is consistent with a broader principle: in patients who survive an acute neurosurgical emergency without overt neurologic injury, rehabilitation should not be deprioritized. The absence of frank weakness can mask substantial functional decline driven by age, comorbidity, and the catabolic stress of sepsis. Limitations of this report include its single-patient design, which precludes causal inference, and the absence of additional quality-of-life instruments (e.g., EQ-5D, SF-36). Future studies incorporating the FIM, Modified Barthel Index, and validated quality-of-life measures across multiple patients are needed to better characterize functional trajectories in this rare scenario.

## Conclusions

Thoracic epidural abscess following an SCS trial is a rare but potentially devastating complication that may present soon after lead removal and progress rapidly despite a deceptively benign neurologic examination. In this patient, timely surgical decompression, targeted antimicrobial therapy for MRSA, and a coordinated inpatient rehabilitation program with strict spinal precautions were associated with the restoration of independent ambulation and ADL function without neurologic decline, as objectively documented by a 21-point gain in the FIM motor sub-score and a 4-point reduction in NRS pain. This case supports prompt recognition of post-SCS infection, multidisciplinary coordination among spine surgery, infectious disease, and PM&R, and routine reporting of standardized rehabilitation outcomes, such as the FIM, to facilitate comparability across future cases.
